# Direct Visualization of Protease Action on Collagen Triple Helical Structure

**DOI:** 10.1371/journal.pone.0011043

**Published:** 2010-06-16

**Authors:** Gabriel Rosenblum, Philippe E. Van den Steen, Sidney R. Cohen, Arkady Bitler, David D. Brand, Ghislain Opdenakker, Irit Sagi

**Affiliations:** 1 Department of Structural Biology, The Weizmann Institute of Science, Rehovot, Israel; 2 Laboratory of Immunobiology, Rega Institute for Medical Research, University of Leuven, Leuven, Belgium; 3 Department of Chemical Research Support, The Weizmann Institute of Science, Rehovot, Israel; 4 Department of Veterans Affairs, Health Science Center, University of Tennessee, Memphis, Tennessee, United States of America; Massachusetts Institute of Technology, United States of America

## Abstract

Enzymatic processing of extracellular matrix (ECM) macromolecules by matrix metalloproteases (MMPs) is crucial in mediating physiological and pathological cell processes. However, the molecular mechanisms leading to effective physiological enzyme-ECM interactions remain elusive. Only scant information is available on the mode by which matrix proteases degrade ECM substrates. An example is the enzymatic degradation of triple helical collagen II fragments, generated by the collagenase MMP-8 cleavage, during the course of acute inflammatory conditions by gelatinase B/MMP-9. As is the case for many other matrix proteases, it is not clear how MMP-9 recognizes, binds and digests collagen in this important physiological process. We used single molecule imaging to directly visualize this protease during its interaction with collagen fragments. We show that the initial binding is mediated by the diffusion of the protease along the ordered helix on the collagen ¾ fragment, with preferential binding of the collagen tail. As the reaction progressed and prior to collagen degradation, gelatin-like morphologies resulting from the denaturation of the triple helical collagen were observed. Remarkably, this activity was independent of enzyme proteolysis and was accompanied by significant conformational changes of the working protease. Here we provide the first direct visualization of highly complex mechanisms of macromolecular interactions governing the enzymatic processing of ECM substrates by physiological protease.

## Introduction

Animal tissues contain an extracellular matrix (ECM) that consists of an intricate network of macromolecules, the primary constituent being collagens. The ECM plays a pivotal role in numerous processes related both to the mechanical properties of a tissue, and also to modulating cellular activity. Under certain conditions, especially during a pathological situation, a rapid and extensive degradation of collagen takes place. In addition to these diseases, which include cancer and autoimmune diseases [1]–[3], collagen degradation also occurs during normal tissue homeostasis. The enzymes active in ECM degradation, members of the matrix metalloproteinase (MMP) family, cooperatively degrade a variety of extracellular proteins, of which collagen is distinguished in being particularly resistant to other proteases due to its tightly packed structure [4], [5].

Members of the MMP family share several conserved protein domains [2], including the catalytic domain, linker, and a C-terminal hemopexin-like domain. MMP-9 is a particularly interesting example of a soluble protease possessing a relatively long glycosylated linker domain [6], [7]. In addition, it contains a fibronectin-like domain, inserted near the active site [8], which is instrumental in collagen/gelatin binding and for gelatinolytic activity, achieved by presenting the scissile bond to the enzyme active site [9], [10]. Each of these protein domains may participate in a particular modification step of the tightly packed triple-helical collagen structure [11], [12].

Native human collagen type-II is susceptible to cleavage by various collagenases (MMP-1, MMP-8, and MMP-13), which cleave at a single position across all three α chains, generating characteristic ¾ and ¼ fragments [13]–[17] which retain the triple-helical structure. It has been shown that MMP-9, which primarily acts only on gelatin, degrades these collagen fragments into small remnant peptides critical in the promotion of autoimmune processes and in acute inflammatory diseases [18]. The molecular mechanisms governing the interactions of this protease with triple helical collagen fragments are far from being understood. As most of available information is derived from indirect kinetic studies, spectroscopy and gel based analyses it is not clear how matrix proteases recognize, bind and process the triple helical structure of intact or fragments of collagen molecules. Computational structural analysis indicates that a triple-helical collagen cannot be accommodated at the active site cleft of a given MMP due to the relatively small size of the enzyme catalytic site [19], [20] (see also Supporting File S1 and Figure S1). Using gel based analysis it has been demonstrated that collagenases locally unwind triple-helical structure of collagen prior to proteolysis [21]. It was suggested that local collagen unwinding may facilitate access of a single chain within the collagen triple-helix to the catalytic site of collagenases [21], [22]. Whereas the importance of the additional fibronectin domain in gelatinases has been studied [11], [23]–[27], the molecular mode by which these important enzymes initially bind and process their triple helical substrates remains to be determined.

To this end, we have designed an Atomic Force Microscopy (AFM) experiment to follow a matrix protease, MMP-9/gelatinase B, during its physiological interaction with triple helical collagen fragments. So far, collagen degradation was studied using bulk methods that provided indirect mechanistic information about collagen processing. Furthermore, only scant information is provided up to date on the gelatinase MMP-9, that plays critical role in pathological processes and proposed as being a good target for drug design [28]. For the first time, we provide direct evidence on this process, obtained by single molecule imaging. Our analysis indicates that the protease-collagen interaction is mediated via a complex molecular mechanism. Initially, the protease diffused along the collagen type II fragment to find and bind the relatively loose tail. Denaturation of the collagen structure at the single-molecule level was monitored and imaged as the reaction proceeded independently of the proteolysis step. The AFM images revealed significant changes in the protease protein domain conformation upon binding to collagen which are postulated to be associated with further deformation of the collagen triple-helical structure to gelatin-like morphologies.

## Results and Discussion

Reliable use of AFM to monitor a biological process is contingent on several conditions: 1.Verification that sample preparation for AFM imaging does not introduce changes of its own. 2. Images of sufficiently high resolution to distinguish the intra-molecular processes of interest. 3. Working conditions and analyses should minimize any possible perturbation by the AFM tip. In order to satisfy the demands of high resolution, we chose to work with semi-dried samples on a mica substrate. This approach has been successful in the past for MMP-9 imaging [6].

The collagen molecules did not undergo denaturation upon binding to the mica (Supporting Figure S2) as also reported by others [29]. The average length of a collagen type-II molecule is 2820 Å. The measured height was 5 Å which was influenced by the force applied on the sample. The measured width represents a convolution with the tip apex, and exaggerates the true molecular width. Our results are in good agreement with previously reported AFM studies [30].

### Single point fragmentation of collagen II

The first step is the characterization of collagen type II physiological fragments generated by incubation of intact collagen II with MMP-8. The collagen monomers show an apparent molecular weight of 130 kDa on reducing SDS-PAGE analysis. Incubation of triple helical collagen monomers with MMP-8 at 30°C resulted in a single position cleavage, producing ¾ and ¼ collagen fragments (Supporting Figure S3A). AFM images of collagen type-II cleaved by MMP-8 reveal that the collagen fragments retained their triple helical structure and remained intact (Supporting Figure S3B). The distribution of fragment lengths shows two dominant subpopulations of 586±7 and 2130±20 Å (mean ± SD). The uncleaved molecules exhibit a length of 2820±10 Å (Supporting Figure S3D).

**Figure 1:**
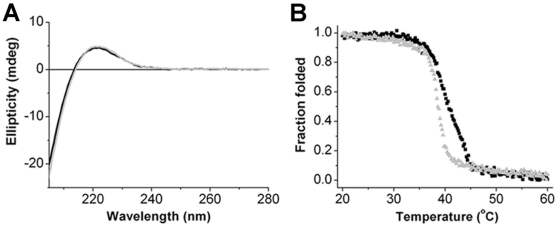
Circular Dichroism spectra and thermal transition curves of intact collagen type II (black) and MMP-8-cleaved collagen (gray). (**A**) CD spectra at a concentration of 60 µg/ml shows the unique triple helical signature at 221 nm with no detectable change between the two samples. (**B**) Thermal transition curves were recorded at 221 nm while the temperature was increased from 20 to 60°C at 0.2°C/min. The melting temperatures, *T_m_^50^*, are 40.7, 38.5°C for intact collagen, and MMP-8-cleaved collagen, respectively.The temperature was increased at slow rate while allowing 1 minute equilibration time before measuring each time-point. These conditions, were maintained in order to allow sufficient time for the system to reach thermal equilibrium before acquisition of CD spectra.

Circular Dichroism (CD) spectroscopy was used in order to further characterize the triple-helical structure of collagen type-II fragments. Figure 1A presents the CD spectra of collagen type-II before and after cleavage with MMP-8 (catalytic amounts of MMP-8 used in this assay are below the detection limit of the CD spectrometer). The spectral transition at 221 nm (Figure 1A) is indicative of the presence of triple-helical structure [23], [24], [31]. No significant change in the ellipticity signal was detected in the CD spectra upon cleavage with MMP-8, indicating that the collagen fragments retain their triple-helical structure under the solution conditions used, in agreement with the AFM observations (Supporting Figure S3B) and with [32].

The thermal stability of the collagen fragments was monitored using a CD thermal denaturation experiment. Attenuation of the 221 nm spectral feature represents loss of the secondary structure content of the triple-helical collagen structure. Plots of percent denaturation vs. temperature are recorded in Figure 1B. The *T_m_^50^* is the temperature at which 50% of the protein is thermally denatured, measured as 40.7°C for intact collagen type-II, whereas following MMP-8 cleavage, the ¾ and ¼ fragments exhibited a reduced *T_m_^50^* of 38.5°C. Our results indicate that a decrease in collagen thermostability is observed upon proteolytic cleavage even though the triple-helical structure is maintained (as shown by the CD spectral analysis). This implies that thermal destabilization occurs at point defects, such as those occuring at the tails created by collagenase cleavage or at regions of different helical stability as demonstrated by the work of Leikin and coworkers [33], [34]. Such structural changes are too subtle to be detected by CD spectra and AFM. The spectral results indicate that thermal denaturation is an irreversible process as noted before. The thermal stability of collagen type II fragments are also analyzed by trypsin digestion followed by gel based analysis (Supporting File S1, Figure S4 and Figure S5) and by AFM (Supporting Figure S6).

### Direct visualization of enzymatic processing of collagen superstructure by MMP-9

**Figure 2:**
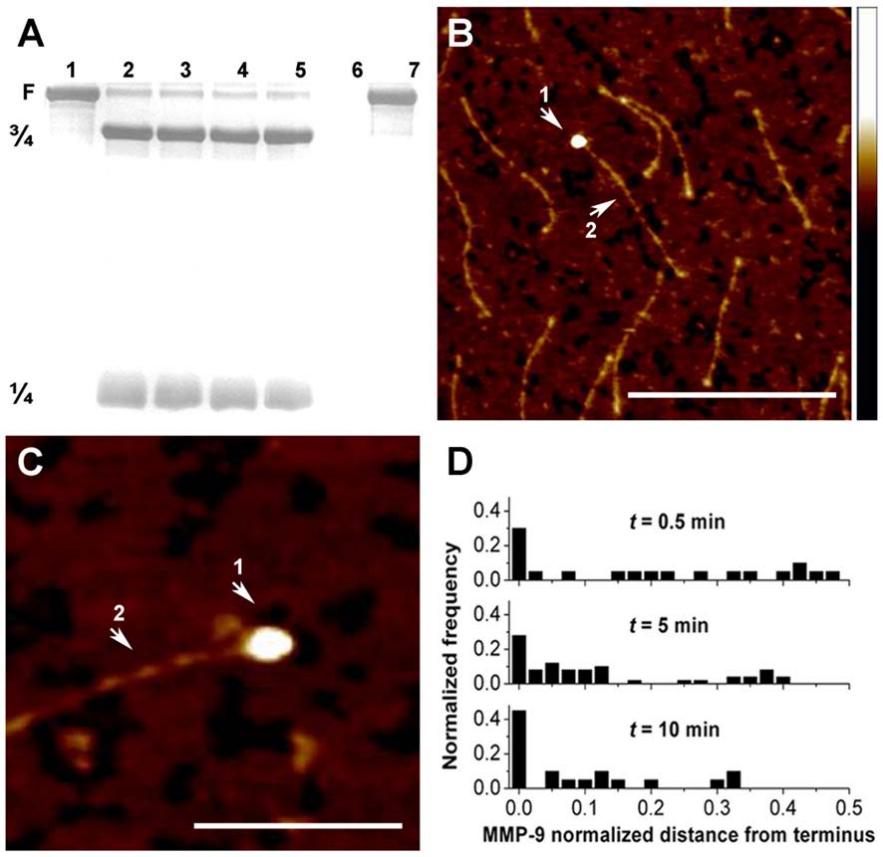
Tails of collagen fragments are observed as loading zones for MMP-9 at short incubation times. (**A**) SDS-PAGE followed by silver staining of intact collagen (lane 1), MMP-8 treated collagen (lane 2), MMP-8 treated collagen incubated with activated MMP-9 for 0.5 min (lane 3), 5 min (lane 4), 10 min (lane 5), 16 h (lane 6), intact collagen incubated 16 h with MMP-9 alone (lane 7). Activated MMP-9 at a final concentration of 20 nM was incubated with the MMP-8-cleaved-collagen at ambient temperature (22°C). (see also Supporting Information on collagen type II). (**B**) AFM tapping mode images of complexes of MMP-9 (arrow 1) and collagen (arrow 2), after 0.5 min incubation. In the majority of the images, MMP-9 is bound at the collagen tails. The scale bar corresponds to 3000Å. (**C**) A different image from the same sample. The scale bar corresponds to 1000 Å. Height scale for all images is indicated by the bar to the right, in which the *Z*-axis ranges from 0 to 50 Å. (**D**) The location of MMP-9 on MMP-8-treated collagen II after incubation time of 0.5 min (*n* = 21), 5 min (*n* = 52), and 10 min (*n* = 22). The *X*-axis represents a normalized distance of MMP-9 from the terminus of the collagen fragment. The scale ranges from 0 to 0.5 (terminus to mid-point). The *Y*-axis is the normalized frequency obtained by dividing the counts by the total population.

Our gel based analysis indicates that degradation of collagen type-II fragments by MMP-9 required long incubation times at ambient temperature (22°C). Ambient temperature conditions were used in order to separate the binding and proteolytic events as well as to maintain the stability of collagen triple helix *in-vitro.* Figure 2A lanes 3–5 show that for short incubation times, MMP-9 did not result in productive proteolysis of collagen type-II. Hence the molecular weight of collagen type-II fragments did not change in the earlier stages of the collagenolytic reaction in the presence of MMP-9 (0.5–10 minutes). Long incubation time eventually results in complete degradation of MMP-8-treated collagen II by MMP-9 (Figure 2A, lane 6). To structurally characterize the early molecular interactions of the protease with collagen type-II fragments we used AFM single molecule imaging experiments. Specifically, we focused on the binding and molecular interactions taking place at the initial stages of the reaction prior to proteolysis which could not be resolved by the gel based analysis (Figure 2A).

AFM images of collagen/MMP-9 mixtures at short incubation times (0.5–10 min) clearly reveal the bound complex and allow analysis of the binding characteristics. Figures 2B–C show the initial binding stages of MMP-9 to collagen. The protease, seen as a globular feature at the collagen tail exhibits characteristic size and overall dimensions which can be compared with our previous measurements of free MMP-9, indicating conformational changes of the working enzyme upon binding to collagen (see below) [6]. Figure 2D shows the changing statistics of bound MMP-9 position with incubation time. The initial diffuse binding distribution became more confined with time, with most of the protease eventually concentrated in the vicinity of the tails. Using spectroscopic analyses Goldberg and coworkers propose the ability of gelatinase A (MMP-2) to diffuse laterally on the gelatin surface [35]. Saffarian *et al*. show that activated collagenase (MMP-1) moves progressively on the collagen fibril following “ bias diffusion mechanism” dependent on collagen proteolysis [36]. Our results provide time dependent snapshots of specific and non-specific MMP-9-collagen type II interactions suggesting that MMP-9 is laterally diffused on collagen prior to binding on preferable substrate sites.

Our single molecule analysis clearly indicates the preference of MMP-9 to bind to the ¾ fragment. For 0.5, 5 and 10 minutes incubation times, we observed 79%, 92% and 100% MMP-9 binding to the ¾ fragment, respectively. Binding of MMP-9 to the ¼ fragment was rarely seen using these experimental conditions and binding to the intact collagen may occur occasionally but overall there was a clear preference for the MMP-9 to bind the ¾ fragment.

### Visualization of collagen denaturation mediated via MMP-9 interactions

**Figure 3:**
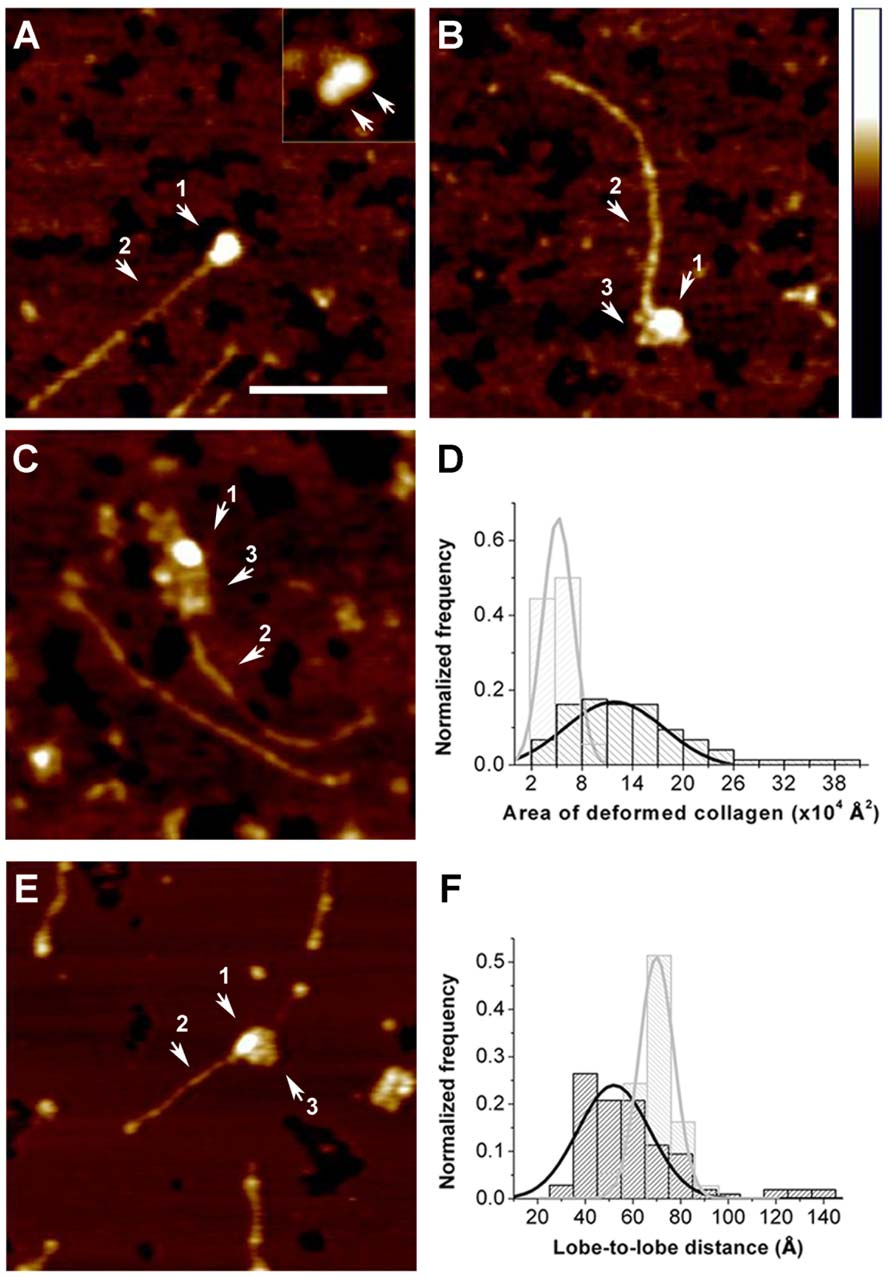
Unwinding of collagen II fragments by MMP-9. MMP-9 (arrow 1) was incubated with MMP-8-treated collagen II (arrow 2) for: (**A**) 0.5 min, (**B**) 5 min and (**C**) 10 min, at ambient temperature (22°C) adsorbed on mica and scanned using tapping mode AFM, showing the ability of MMP-9 to unwind (arrow 3) collagen tails in solution. The scale bar corresponds to 1000 Å. The insert in panel **A** is collagen-free MMP-9 zymogen in the extended conformation where the two terminal domains are resolved (see arrows). Height scale for all images is indicated by the bar to the right, in which the *Z*-axis ranges from 0 to 50 Å. (**D**) Surface area of MMP-9-degraded collagen II after an incubation time of 0.5 min (gray, *n* = 21) and 5 min (black, *n* = 52). Surface area was calculated as indicated in Supporting file S1. (**E**) The catalytically inactive MMP-9(E402A) mutant, was incubated with MMP-8-treated collagen for 10 min, adsorbed to mica and scanned, showing the ability of this catalytically inactive mutant to unwind collagen. Scales are identical to panels A–C. (**F**) Lobe-to-lobe distance of MMP-9 in its collagen-bound (black, n = 107) and free (gray, *n* = 39) state. The *Y*-axis of all histograms is the normalized frequency obtained by dividing the counts by the total population. Although the collagen-bound and collagen-free MMP-9 molecules were scanned using different cantilevers (2 vs. 5 N/m) and different routine of sample preparation, these factors should not strongly affect their lobe-to-lobe distances. The reported statistics consists only on lobe-to-lobe distances that were clearly distinguished at the XZ cross-section as two peaks separated by a lower saddle-point.

The AFM images at different incubation times can furthermore be used to monitor the extent of denaturation of the triple-helical structure of the collagen tail by MMP-9 as the reaction progresses. Figures 3A–C show a series of images after 0.5, 5, and 10 minutes of incubation of the collagen fragments with MMP-9. The predominant molecular event as captured at 0.5 min incubation time appears to be complex formation of collagen and MMP-9 (Figure 3A). After 5 min incubation time (Figure 3B), features appeared at the MMP-9 binding site which are assigned to the formation of a small area of denatured collagen. The area of these patches dramatically increased after an incubation time of 10 min (Figure 3C) possibly due to partial degradation of the collagen molecule. Such pronounced structural deformation of the collagen was statistically evaluated by measuring the area of denatured collagen molecules following interaction with MMP-9 (Figure 3D - additional details on the characteristics of denatured collagen images are given in Supporting File S1 and Supporting Figures S7, S8 and S9). At ***t*** = 0.5 min, enzymatic denaturation of the collagen structure was fairly moderate and the calculated molecular area (see Supporting File S1 and Supporting Figure S7 for details) exhibited an average value of 4.8±2.2×10^4^ Å^2^. At ***t*** = 5 min the deformed area grew by a factor of 3 to an area of 14±7.5×10^4^ Å^2^. Reliable statistical analysis could not be performed for samples measured at ***t*** = 10 min due to considerable amount of scattered “background material” on the mica surface, much of which likely arises from digested collagen which separated from the parent collagen fragment. Nevertheless, denaturation of collagen type-II fragments by MMP-9 was clearly detected at the tails of the collagen molecules. Recently, Coletta and coworkers rationalized the poor affinity of MMP-9 to collagen IV by the necessity of MMP-9 to induce a conformational change on its substrate to expose the binding site [37]. Our findings provide direct evidence for this hypothesis and further reveal that this phenomenon is localized at the collagen tail. Further evidence that the enzymatic activity occurred at the tail of the collagen molecule is the fact that we could not detect the 1/4 fragment after exposure to MMP-9. Since the tails represent a much larger fraction of the 1/4 as compared to the 3/4 fragment, these smaller fragments were presumably were more effectively digested to the point of complete decomposition.

### Independence of MMP-9 collagen denaturation activity and peptide hydrolysis activity

Our SDS-PAGE and AFM analyses (Figure 2) indicate that denaturation of collagen type-II fragments occurs prior to proteolysis. As a further test of this two-staged action we performed an AFM experiment with a catalytically inactive MMP-9. In this mutant the catalytic Glu402 is mutated to Ala. This mutant does not cleave collagen type-II even after full denaturation [38], [39]. Figure 3E demonstrates that MMP-9(E402A) was capable of denaturing a collagen type-II ¾ fragment at the tail similar to the action of wild type MMP-9. CD characterization and AFM statistical analysis is provided in Supporting File S1 and Supporting Figures S10 and S11. These results show conclusively that the denaturing activity of MMP-9 is independent of collagen proteolysis. This finding is consistent with other studies that examine limited proteolysis and biochemical analyses of the inactive collagenases MMP-1(E200A) and MMP-8(E198A) that locally unwinded collagen type I [11], [21], [22]. Similar studies are also reported for MMP-2 and collagen type I [23], [27]. Remarkably, our AFM images provide direct visualization of collagen deformation localized at MMP-9 functional sites of collagen type II fragments.

### Collagen denaturation is mediated by MMP-9 conformational changes

Figure 3A inset shows a single molecule image of free (unbound) MMP-9 zymogen. Full length MMP-9 exhibits an elongated dumbbell like structure consisting of two terminal domains (see arrows) and an extended linker domain [6]. Statistical analysis of the lobe-to-lobe distance between the two terminal domains indicates that the relatively long enzyme linker domain (designated OG domain) is flexible and may adopt two main conformations, namely extended and contracted. Here, single molecule analysis of the lobe to lobe distance distribution of MMP-9 in complex with collagen shows that the enzyme adopts the contracted conformation when bound to collagen. The statistical analysis displayed in Figure 3F shows that the lobe-to-lobe average distance of collagen-bound MMP-9 (60±20 Å) was significantly different from the free MMP-9 (74±10 Å [6]) (*t*-test of unequal variances, *P*<0.001). These results suggest that the observed conformational change of MMP-9 is associated with the denaturation of the triple-helical structure of collagen. Such functional protein conformational changes of MMP-9 during collagen processing were previously predicted by Overall and Butler [40] based on the solution structure of MMP-9 zymogen [6]. Remarkably, our single molecule images provide direct evidence that MMP-9 changes its free state elongated conformation to a more gloubular conformation upon binding of collagen.

This work provides direct visulaization of the molecular mechansims and the mode by which a MMP-9 recognizes and processes triple helical fragments of collagen type II. Using single molecule imaging analysis enabled us to directly visualize the overall molecular interaction of MMP-9 with collagen type II fragments. This includes the identification of preferential substrate binding sites of the enzyme, collagen deformation by MMP-9 and quantification of MMP-9 conformational changes. These mechanistic and structural insights on functional deformation of collagen by MMP-9 activity could not be detected using ensemble measurements.

Initially, MMP-9 is randomly distributed along the collagen fragment (Figure 2D), becoming localized at the tail as the reaction progresses. This may indicate that the collagen molecules become more relaxed with time promoting higher affinity to the enzyme, particularly at the tails. An alternative interpretation is that the enzyme inches along the collagen, scanning for denatured and damaged regions or its cleavage recognition sequence. The ability of MMPs to move along collagen fibrils has been investigated [36], and it is suggested that MMPs may utilize a Brownian ratchet mechanism for “biased diffusion”. Direct visualization of the time-dependent accumulation of MMP-9 population at the tails provide convincing proof of this concept, emphasizing the ability of MMP-9 to diffuse along to a point where binding is preferred e.g. the relaxed tails of the ¾ collagen fragment. The flexibility of the enzyme linker domain [6] may contribute to this sliding ability of MMP-9 as suggested by Overall and Butler [40]. Collagen denaturation at the tail by MMP-9 is observed as the reaction progresses with time (Figures 3B–C), manifested by an increased area of deformed collagen. Remarkably, this denaturation activity results in the formation of gelatin-like regions. This mechanistic aspect distinguishes MMP-9 from other collagenases where local unwinding (at the middle of the collagen molecule) appears to be synchronized with the cleavage event [21]. In addition, the preferential binding site and cleavage sites of MMP-9 at the collagen tails differs from collagenases that cleave internal triple-helical regions at the interface of tight and loose helical structures [41]. Thus, being a gelatinase, MMP-9 denatures the triple helical structure of collagen into gelatin-like morphologies which could be instrumental in promoting proteolysis by this enzyme.

Degradation of collagen type-II fragments by synergistic action of collagenases and MMP-9 is utilized for collagen turn-over and for the production of peptides critical in the establishment and perpetuation of autoimmune processes and in acute inflammatory diseases [18]. Here we showed that the triple helical structure of collagen type II remained intact after single point cleavage by the collagenase (MMP-8). Our experiments demonstrated that, *in vitro*, the triple helix of the collagen fragments has a decreased stability at 37°C and became fully denatured above 39°C (Supporting File S1 and Supporting Figures S4 and S5). However, complete collagen II denaturation is not likely to take place *in vivo*, as it is stabilized by interaction with many other glycoproteins in a dense matrix and by the presence of intact telopeptides and cross linking [5]. Therefore, the results reported here argue that *in vivo* MMP-9 may further destabilize triple-helical based collagen structures by the mechanism exposed in Figure 3. We postulate that binding of MMP-9 to collagen may adopt a sandwich conformation in which the collagen molecule closely interacts with the exosites of the enzyme including its long linker domain. This mode of enzyme-collagen interactions may mediate the observed local denaturation of the relatively loose collagen tail. In support of this concept, MMP-9 mutant lacking the long linker domain hydrolyzed single chain collagen peptides and gelatin in vitro. This further suggests that the observed “sandwich conformation” of MMP-9 which brings the two terminal domains together may be critical for cleaving the denatured triple-helical collagen substrate. Our results demonstrate that the complex reaction of collagen proteolysis is mediated by a sequence of structural and dynamic events taking place in both the enzyme and the collagen substrate. In addition, the AFM images suggested that MMP-9 (gelatinase B) deforms the collagen triple helical structure to a gelatin-like structure, a natural substrate of this enzyme, prior to proteolysis.

MMP-9 is produced and released by neutrophil granulocytes during acute inflammation in the joint. The cooperative molecular mode by which this protease catalyzes collagen breakdown, described herein, offers direct insights into the versatile interactions of matrix proteases with ECM.

## Materials and Methods

All solutions were prepared with ultrapure milli-Q (Millipore) water, and were subjected to 0.22 µm filtration prior to use. AFM experiments facilitated a direct visualization of human collagen type-II fragments during processing by MMP-9. Homogenous protein samples were prepared for AFM imaging. Protein samples were further analyzed by gel electrophoresis prior to AFM imaging. Atomically-flat mica provided a featureless substrate which allowed facile and high-quality imaging. The applied concentrations were chosen to yield an adequate surface coverage with ten or more isolated collagen molecules per µm^2^ (Supporting Figure S2).

### Matrix Metalloproteinases

Recombinant pro-MMP-9 was expressed by infection of Sf9 insect cells with a baculovirus carrying the cDNA of human proMMP-9 [39]. Liter quantities of cell culture fluids were centrifuged, filtered and purified to homogeneity by gelatin-Sepharose chromatography [42]. The material was extensively dialyzed in 100 mM Tris pH 7.4, 100 mM NaCl, 10 mM CaCl_2_ (buffer C). The monomeric form was separated from other higher oligomeric forms by glycerol gradient sedimentation. A sample (0.2 mg) of purified pro-MMP-9 was layered onto four polyallomer tubes containing 10–45% glycerol gradient (prepared in GradientMaster BioComp™) in buffer C. The tubes were then centrifuged in a SW41 rotor at 37,000 rpm, 63 h, 4°C. The gradient was then fractionated to 0.5 ml samples that were assayed for the presence of monomeric and other oligomeric structures by gelatin zymography [42]. Fractions containing homogenous monomeric structures were pooled and dialyzed against buffer C to remove excess glycerol. Activation of pro-MMP-9 was performed by incubation with either 1 mM p-aminophenyl mercuric acetate (APMA) for 1 hr at 37°C or by the recombinant active catalytic domain of MMP-3 (Merck-Calbiochem) at 1∶100 molar ratio for 1.5 hr. Pro-MMP-8 (R&D Systems) was APMA-activated and used for collagen pretreatment. All MMPs were tested for their activity using fluorogenic substrate [43]. Conventional SDS-PAGE analysis was used to follow the apparent molecular weight of activated vs. zymogenic MMP-8 and -9.

### Collagen preparation

Collagen type-II was extracted from fetal bovine cartilage using a modification of the methods employed by Miller *et al.* [44]. Lyophilized collagen was solubilized in ice-cold 0.6% glacial acetic acid for 24 h at 4°C. The final concentration of the stock solution was 0.1 mg/ml. Flash-frozen aliquots were stored at −20°C. Prior to use 10 µl collagen solution was mixed with 5 µl 0.2 M Tris (base), 1.5 µl 10× buffer C and 83 µl 1× buffer C (100 mM Tris pH 7.4, 100 mM NaCl, 10 mM CaCl_2_).

### Collagen cleavage

Collagen type-II (0.01 mg/ml, 10 µl) was first incubated with activated MMP-8 at a final concentration of 30 nM at 30°C for 16 h. Activated MMP-9 at a final concentration of 20 nM was incubated with the MMP-8-cleaved-collagen for 0.5, 5, 10 min at ambient conditions (22°C). Collagen cleavage was verified by SDS-PAGE followed by the silver stain procedure. Intact collagen samples not subjected to boiling prior to gel electrophoresis remained at the upper part of the resolving gel, whereas boiled samples migrated as a 130 kDa band. This indicated that thermal unwinding of the triple-helical structure enables single α-chains to be resolved. The apparent molecular weight of a single α-chain is 130 kDa, and therefore we assume 390 kDa for the triple-helical collagen. Under this assumption, the molar ratio between MMP-9 to collagen is ∼1∶1.

### Atomic Force Microscopy imaging

The samples were prepared by incubating 2 µl mixtures on freshly cleaved mica for 3 min in a sealed compartment at room temperature. The mica was then washed with 1 ml ultrapure milli-Q water and dried under nitrogen flow. Imaging was performed using a Multimode atomic force microscope (MMAFM Veeco Metrology Division, Santa Barbara, CA, USA) equipped with an E-scanner, with a working range of up to 14×14 µm^2^. Samples were imaged in air using Tapping Mode™. The amplitude set point was adjusted to the maximum value that gave a stable trace. High-resolution images of biological samples in air were obtained using “Tetra tip” – OMCL-AC240TS probes (Olympus Corp., Tokyo, Japan). These probes have a resonant frequency of ca. 70 kHz, a force constant of ca. 2 N/m, and a radius of curvature of 7 nm or less. The sizes of the protein molecules were determined from cross-sectional analysis, or a custom-designed area measurement (Supporting File S1).

Initially, the intact and MMP-8-cleaved collagens were scanned separately in order to characterize their end-to-end length. Subsequently, images were acquired on samples containing mixtures of all three forms as result of incubation with the different enzymes for varying times. Images were produced using NanoScope 7.2 software (Veeco Instruments Inc). For detailed description of the statistical analysis, see Supporting File S1.

### Circular Dichroism Spectroscopy

CD data were recorded using an Applied Photophysics – Chirascan spectrometer using a 0.1 cm path length quartz cell. Samples of collagen type-II at a final concentration of 60 µg/ml (1.5 µM) either with or without MMP-8 (3 µg/ml or 60 nM) were measured. The catalytically inactive mutant MMP-9(E402A) [38] was supplemented at a final concentration of 0.01 mg/ml (120 nM) to another sample that contained collagen and MMP-8 at the indicated concentrations. All samples were incubated at 30°C for 16 h to allow complete collagen cleavage (by MMP-8) as verified by silver-stained SDS-PAGE analysis. For detailed description of CD measurements, see Supporting File S1.

## Supporting Information

File S1 (0.05 MB DOC)

Figure S1 Computational protein-protein docking of a triple helical peptide (sticks) to the activated form of MMP-9 catalytic domain. A view along the collagen axis (top), and 90° rotation for a view down the collagen axis (bottom). Collagen triple helical peptide [3] was docked to the catalytic domain of MMP-9 [4], where the pro-peptide domain (Val29-Arg106) was excluded. The catalytic zinc ion is represented by a red sphere. The three fibronectin repeats are shown to the left (top), or at the back (bottom).
(0.22 MB DOC)

Figure S2 AFM images of monomeric collagen type II. Full length monomeric collagen type II molecules were imaged using AFM. The collagen solution was diluted in order to single out individual monomers of collagen type II. The protein solution was adsorbed on mica and scanned in tapping mode. The height scale is indicated by the bar to the right, in which the Z-axis ranges from 0 to 30 Å (dark to light).
(0.22 MB DOC)

Figure S3 Full-length collagen type II cleavage by MMP-8. (A) SDS-PAGE of intact collagen (lane 1, marked F), and MMP-8 produced collagen fragments (lane 2, marked 3/4 and 1/4). Proteolysis of collagen type II by MMP-8 was conducted at 30°C (see supporting Information for collagen thermal stability under these reaction conditions). (B) A mixture of intact and cleaved collagen (1/4 and 3/4 fragments) was adsorbed on mica and scanned by tapping mode. Intact collagen is marked with F and the collagen fragments are marked 1/4 and 3/4. This picture was generated after distinguishing full-length collagen from the 1/4 and 3/4 fragments produced by complete MMP-8 cleavage, in separate AFM experiments. (C) The MMP-8 treated collagen was heat-denatured at 70°C showing complete loss of structural features. Insert - magnification of denatured collagen. Height scale for all images is indicated by the bar to the right, in which the Z-axis ranges from 0 to 20 Å (dark to light). (D) End-to-end collagen length histogram of intact, 3/4 and 1/4 fragments (mean lengths 2820, 2130 and 586 Å, respectively). The values were extracted from the AFM images (n = 151). The Y-axis is the normalized frequency obtained by dividing the counts by the total population.
(0.18 MB DOC)

Figure S4 Thermal stability assay of intact collagen type II and collagen type II fragments used in AFM experiments. The basis for the assay is that one can leverage collagen's resistance to proteolytic digestion by enzymes other than collagenase. Native collagen will not be degraded by trypsin or chymotrypsin. However, denatured collagen is no longer resistant to this type of digestion, and will become susceptible to cleavage by trypsin and chymotrypsin. Experimental conditions were slightly modified from reference [5]. Briefly, Bovine type II collagen digested at room temprature overnight with preactivated MMP-8 (1∶100) (obtained from Dr. Karen Hasty). Digested or non-digested samples heated to the designated temperature for 30 seconds, then digested with trypsin for 5 minutes. All reaction stopped with EDTA. Samples run on 7.5% gel. The 3/4 and 1/4 collagen fragments are clearly visible in the MMP-8-treated samples (designated as TCA and TCB in the key presented in the right panel). Significant collagen degradation by MMP-8 starts at 35°C (left panel). Noteworthy, the AFM experiments were conducted at lower temperature using physiological buffer. The designated 3/4 fragment was analyzed by single molecule AFM imaging statistical analysis.
(0.79 MB DOC)

Figure S5 Thermal stability of intact collagen. Since the stability of collagen type II preparations may vary, we include here the collagen thermal stability test assay of the original intact collagen type II preparation used for the AFM experiments. 50 µg of type II collagen (1 mg/ml in digest buffer) incubated at the given temperature for 1 minute, followed by a 4 minute digest with 1 µg of trypsin. The reaction is then stopped with 5 µg of trypsin inhibitor plus 1 µl of 0.5 M EDTA. The digest is mixed with Laemmli buffer and run on a 7.5% gel. This collagen preparation is stable up to 42°C, as indicated by persistence of the bands.
(0.86 MB DOC)

Figure S6 AFM control image of collagen type II fragment (treated with MMP-8) after 1 hour incubation at 37°C. The collagen fragments retain their intact structure as can be clearly detected by this image. Substantial collagen deformation could be detected only after incubation with MMP-9 as reported in text.
(0.06 MB DOC)

Figure S7 AFM image of 3/4 collagen II fragment treated with MMP-9 for 5 minutes. The height scale is indicated by the bar to the right, in which the Z-axis ranges from 0 to 50 Å (dark to light). Insert - the selected unraveled part of collagen is shown at the right top corner of the image. Magnification 2× of the full presented AFM image.
(0.36 MB DOC)

Figure S8 Simultaneously acquired height and phase AFM images of MMP-8-cleaved collagen fragment. (A) Height image of a collagen fragment shows that the upper tail (see arrow) is broadened. The height scale is indicated by the bar to the right, in which the Z-axis ranges from 0 to 3.5 Å (B) The phase image shows that this area appears darker (see arrow). The height scale is indicated by the bar to the right, in which the Z-axis ranges from 0 to 4 degrees.
(0.14 MB DOC)

Figure S9 Unwinding of mica-adsorbed collagen II fragments by MMP-9. MMP-8-treated collagen II fragments (arrow 2) were adsorbed directly on mica, supplemented with activated MMP-9 (arrow 1) and inspected for unwinding activity (arrow 3). The different stages of the reaction are shown by representative images: (A) tail binding, (B) tail unwinding (C) gross tail unwinding. The samples were scanned using tapping mode AFM. The ability of MMP-9 to unwind insoluble collagen tails when adsorbed to the mica surface is clearly demonstrated. Height is indicated by the bar to the right, in which the Z-axis ranges from 0 to 40 Å.
(0.12 MB DOC)

Figure S10 Circular Dichroism spectra and thermal transition curves of MMP-8-cleaved collagen (black), and MMP-8-cleaved collagen supplemented with the MMP-9 inactive mutant MMP-9(E402A) (gray). (A) CD spectra at a collagen concentration of 60 µg/ml shows the unique triple helical signature at 221 nm with no detectable change between the two samples. (B) Thermal transition curves were recorded at 221 nm while the temperature was increased from 20 to 60°C at 0.2°C/min. The differences in melting temperatures, Tm^50^, are insignificant with 38.5°C between both the MMP-8-cleaved collagen (black), and MMP-8-cleaved collagen supplemented with MMP-9(E402A) (gray).
(0.15 MB DOC)

Figure S11 Local denaturating activity of collagen type II fragment by the latent MMP-9(E402A) mutant at 10 minutes incubation time. Right panel shows statistical analysis of the deformed collagen width interfacing with MMP-9 (Black is wild type MMP-9 and gray is MMP-9(E402A) latent mutant), which scales with the total deformed area. Comparison of the mean width with that of the wild type after 10 minutes incubation time reveals that the area resulting from interaction with mutant is approximately 70% that for the wild type. Local collagen deformation by MMP-9(E402A) is significant but less than observed for the wild type enzyme (n = 97 and 23 for the wild type and MMP-9(E402E), respectively). The left panel of the Figure shows images of collagen type II unwound by MMP-9(E402A) (see also Fig. 3E in main text).
(0.18 MB DOC)
