# Supplementary material for: Direct Visualization of Protease Action on Collagen Triple Helical Structure
Source: PLoS One. 2010 Jun 16;5(6):e11043. doi: 10.1371/journal.pone.0011043 (PMC2886829; doi:10.1371/journal.pone.0011043)
Supplement: File S1 — (0.05 MB DOC) [file pone.0011043.s001.doc]

**Supporting File S1**

**Direct Visualization of Protease Action on Collagen Triple Helical Structure**

Gabriel Rosenblum, Philippe E. Van den Steen, Sidney R. Cohen, Arkady Bitler, David D. Brand, Ghislain Opdenakker and Irit Sagi

***Computational protein-protein docking***

#### The MMP-9-collagen complex was examined using the protein-protein docking package 3D-Dock [1,2]. The crystal structures used for docking were PDB ID: 1BKV [3] corresponding to a triple helical peptide and PDB ID: 1L6J [4] with residues 107-444 corresponding to the activated MMP-9, (the pro-domain is absent and the catalytic domain and the three fibronectin repeats are present). A global scan of rotational and translational space was preformed using Fourier transform while the grid spacing was 0.7 Å. The output complexes were ranked according to surface complementarities and electrostatic characteristics. As shape complementarity is not sufficient to discriminate between large numbers of docked complexes, the structures resulting from the global search were re-ranked with empirically derived potential using the static likelihood of residue-residue contacts across the interface of the complexes. Finally, biochemical and structural knowledge was used to filter solutions; complexes were discarded if the catalytic zinc ion of MMP-9 was more than 10 Å away from the collagen-like peptide. Accommodation of the collagen-like peptide in the deep groove that contains the catalytic zinc of MMP-9 was not observed in any of the calculated complexes.

***Thermal stability of intact collagen type II and collagen type II fragments***

We analyzed the thermal stability of the examined collagen molecules under the reaction conditions used for AFM imaging. Specifically, we digested intact and MMP-8-treated collagen II with trypsin (and chymotrypsin) at various temperatures, including 22°C as control temperature, and 29, 32, 35, 37, 39 and 41°C as experimental conditions (supporting Fig. S4 and Fig. S5). We also included an undigested control sample in the analysis. In line with our other data we observed similar thermal stability from 22° C till 35° C. At 37 °C the trypsin-resistant intact collagen II fraction remained stable, whereas the collagenase-treatment resulted in partial trypsin sensitivity. At and above 39 °C , thermal denaturation occurred and, as expected, trypsin digested the complete pool of molecules, irrespective of the presence of collagenase.

***Image processing and data acquisition***

Unwinding of the MMP-8-treated collagen was quantified by measuring the area of regions which were associated with unraveled collagen segments. Perusal of the images clearly showed that after MMP-9 treatment, many of the collagen molecules were partially digested/unwound as indicated by diffuse patches at one terminus. Geometric areas of these patches were determined by applying a MATLAB (MATLAB R2007a, The MathWorks, Inc., Natick, MA) program developed in house for this purpose. Regions of interest were selected from images of the MMP-9 – treated collagen, interactively based on the criteria of: (1) being contiguous with a collagen strand, and (2) being associated with an MMP-9 molecule. Supporting Fig. S7 shows one such image, with a collagen molecule in the center exhibiting such a diffuse patch with MMP-9 at one end. The program included a module which allowed processing of a large set of images in batch processing mode.

The basic features of the program include:

1). Opening image, automatic reduction of intensity (height) histogram for ease of viewing, and low-pass filtering. The operator then selects regions for further analysis.

2). Image transformation to ‘black-white’ (binary) image and cleaning it from the small background objects (with area less than a threshold value – 50 pixels). A typical selected region after these procedures is presented in the insert of Supporting Fig. S6.

The object area (area of unraveled collagen) is calculated as the total number of pixels constituting the object. These dimensionless values are then transformed into area in units of Å^2^.

***Distinction between mechanical properties of MMP-9-treated tails and central triple helical region of collagen fragments is revealed by phase AFM images***

The application of tapping mode AFM allows qualitative assessment of the sample mechanical properties. The interaction of probe and sample under driven oscillation leads to changes in the phase of oscillation resulting in a phase lag between drive and detected cantilever oscillations. Energy dissipation due to factors such as stickiness (adhesion) can lead to significant changes in phase. Therefore, a comparison of topography and phase images obtained in the tapping mode yields important information about sticky regions on the object of interest. This application proved useful for investigating the interaction of collagen with MMP-9, which transforms MMP-8-treated collagen II into a sticky gel-like phase. The degree of stickiness is dependent on the extent of the chemical change, and appears with varying contrast at different locations along the molecule. The topography and corresponding phase images shown in supporting Fig. S8 demonstrate this effect. The collagen tails in the topography image are broadened and smeared suggesting that these parts are already converted to the gel-like phase. The corresponding phase image demonstrates an additional contrast, suggesting that the degree of stickiness is greater on the upper tail and hence the reaction is more complete relative to the lower tail. In contrast, the central part does not exhibit phase contrast which is consistent with the fact that structure is retained there. This is also reflected in the topographic image where two helical strands of the collagen are clearly detected.

***AFM images reveal the ability of MMP-9 to unwind non-soluble collagen***

In this reference experiment, MMP-9 was added to surface-adsorbed-collagen (whereas in the experiments mentioned in the main text, the collagen digestion was in solution). As observed in Supporting Fig. S9, MMP-9 shows triple-helicase activity under these conditions as well. This reference experiment shows that even on these surface-bound molecules, unwinding is a result of MMP-9 activity localized near or at the collagen tails. This activity is physiologically relevant since collagen monomers assemble in the extracellular matrix to form insoluble fibers.

***Circular Dichroism analysis of the ability of the catalytically inactive mutant, MMP-9 (E402A) to process collagen***

The catalytically inactive MMP-9 (MMP-9(E402A)), where the catalytic Glu402 is mutated to Ala) was checked for its unwinding activity under identical conditions as the wild type.

Since this mutant does not appear to cleave collagen [6], it was inspected for its ability to induce global changes on collagen using CD (Supporting Fig. S10). A significant change could not be detected in the CD spectra and thermal transition curves of collagen fragments prior and following the addition of MMP-9(E402A). This result is in agreement with the results of the catalytically inactive MMP-1(E200A) and MMP-3(E202A) which also did not show a significant change in *T_m_* [7].

**References**

1. Gabb HA, Jackson RM, Sternberg MJ (1997) Modelling protein docking using shape complementarity, electrostatics and biochemical information. J Mol Biol 272: 106-120.

2. Moont G, Gabb HA, Sternberg MJ (1999) Use of pair potentials across protein interfaces in screening predicted docked complexes. Proteins 35: 364-373.

3. Kramer RZ, Bella J, Mayville P, Brodsky B, Berman HM (1999) Sequence dependent conformational variations of collagen triple-helical structure. Nat Struct Biol 6: 454-457.

4. Elkins PA, Ho YS, Smith WW, Janson CA, D'Alessio KJ, et al. (2002) Structure of the C-terminally truncated human ProMMP9, a gelatin-binding matrix metalloproteinase. Acta Crystallogr D Biol Crystallogr 58: 1182-1192.

5. Sieron AL, Fertala A, Ala-Kokko L, Prockop DJ (1993) Deletion of a large domain in recombinant human procollagen II does not alter the thermal stability of the triple helix. J Biol Chem 268: 21232-21237.

6. Van den Steen PE, Van Aelst I, Hvidberg V, Piccard H, Fiten P, et al. (2006) The hemopexin and O-glycosylated domains tune gelatinase B/MMP-9 bioavailability via inhibition and binding to cargo receptors. J Biol Chem 281: 18626-18637.

7. Chung L, Dinakarpandian D, Yoshida N, Lauer-Fields JL, Fields GB, et al. (2004) Collagenase unwinds triple-helical collagen prior to peptide bond hydrolysis. Embo J 23: 3020-3030.
