# Supplementary figures and images for: Direct Visualization of Protease Action on Collagen Triple Helical Structure
Source: PLoS One. 2010 Jun 16;5(6):e11043. doi: 10.1371/journal.pone.0011043 (PMC2886829; doi:10.1371/journal.pone.0011043)

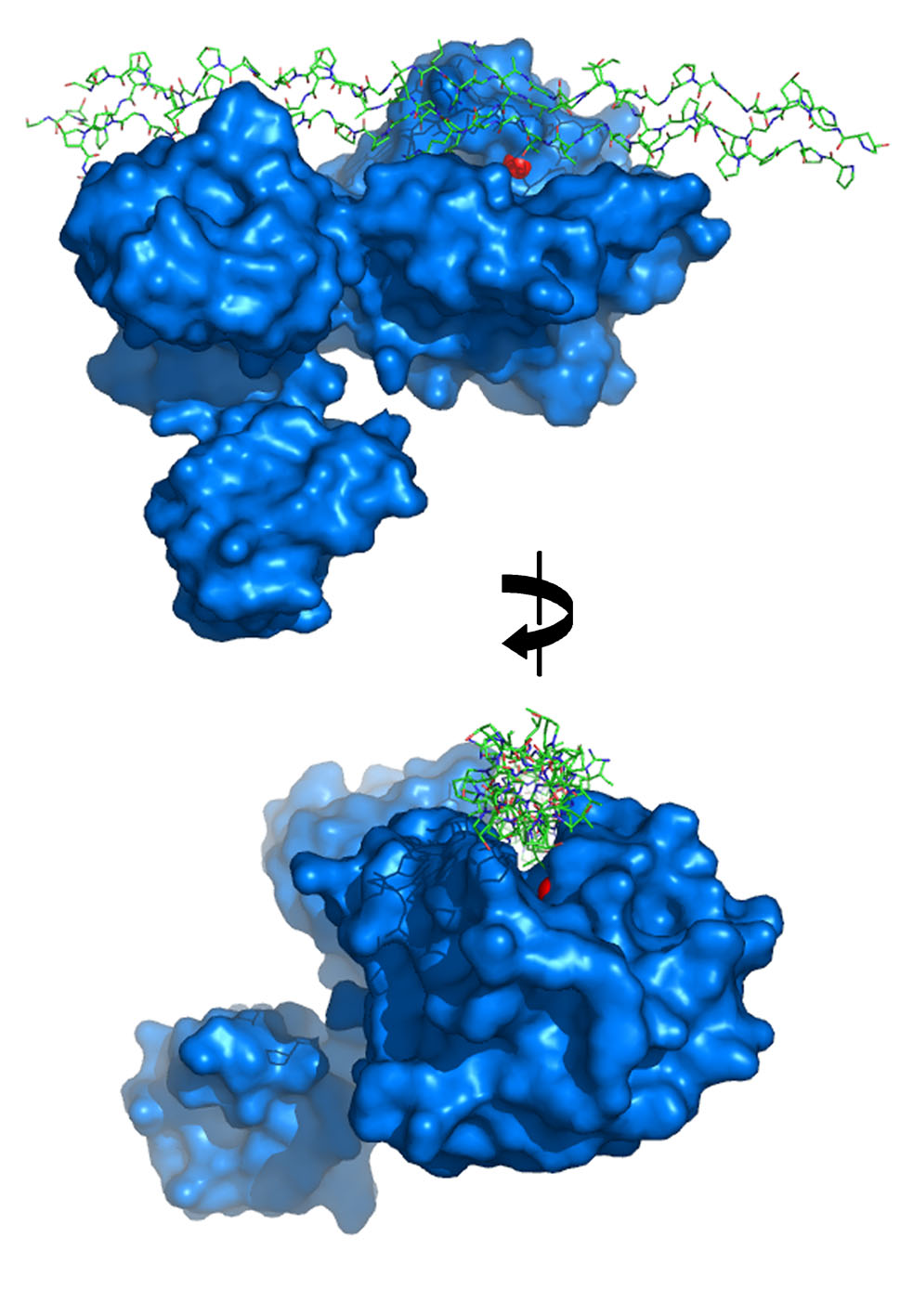

Supplement: Figure S1 — Computational protein-protein docking of a triple helical peptide (sticks) to the activated form of MMP-9 catalytic domain. A view along the collagen axis (top), and 90° rotation for a view down the collagen axis (bottom). Collagen triple helical peptide [3] was docked to the catalytic domain of MMP-9 [4], where the pro-peptide domain (Val29-Arg106) was excluded. The catalytic zinc ion is represented by a red sphere. The three fibronectin repeats are shown to the left (top), or at the back (bottom). (0.22 MB DOC) [file pone.0011043.s002.doc]

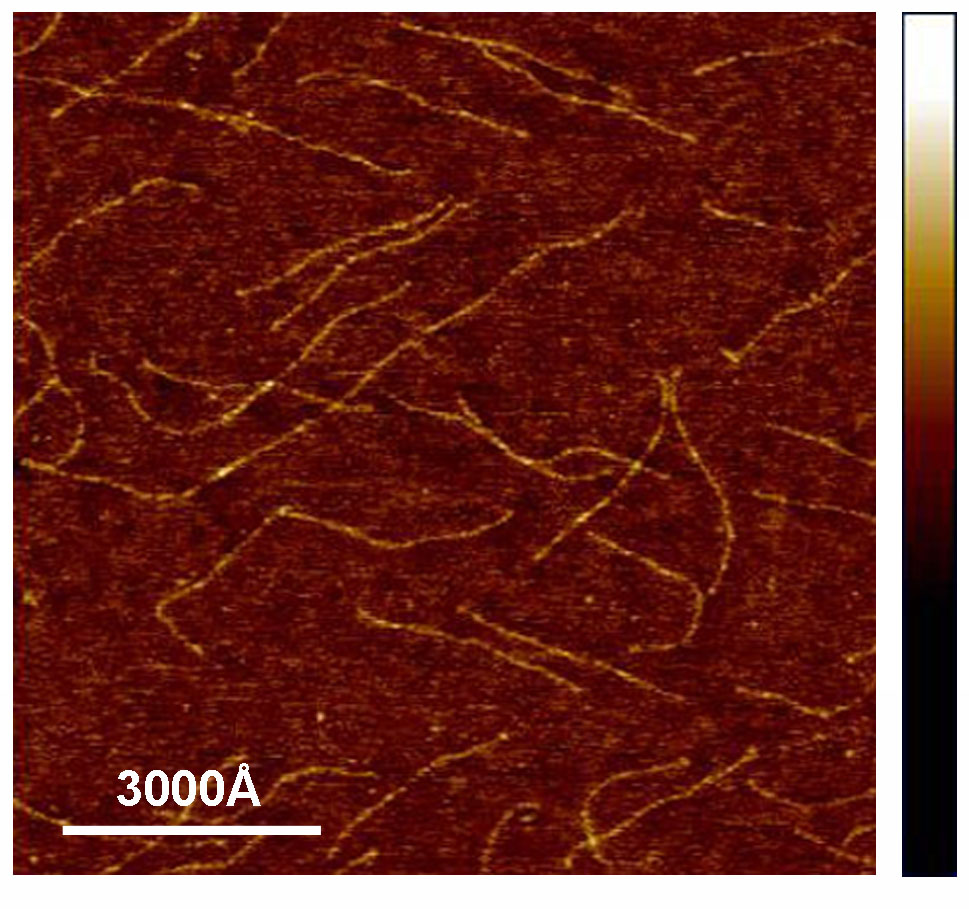

Supplement: Figure S2 — AFM images of monomeric collagen type II. Full length monomeric collagen type II molecules were imaged using AFM. The collagen solution was diluted in order to single out individual monomers of collagen type II. The protein solution was adsorbed on mica and scanned in tapping mode. The height scale is indicated by the bar to the right, in which the Z-axis ranges from 0 to 30 Å (dark to light). (0.22 MB DOC) [file pone.0011043.s003.doc]

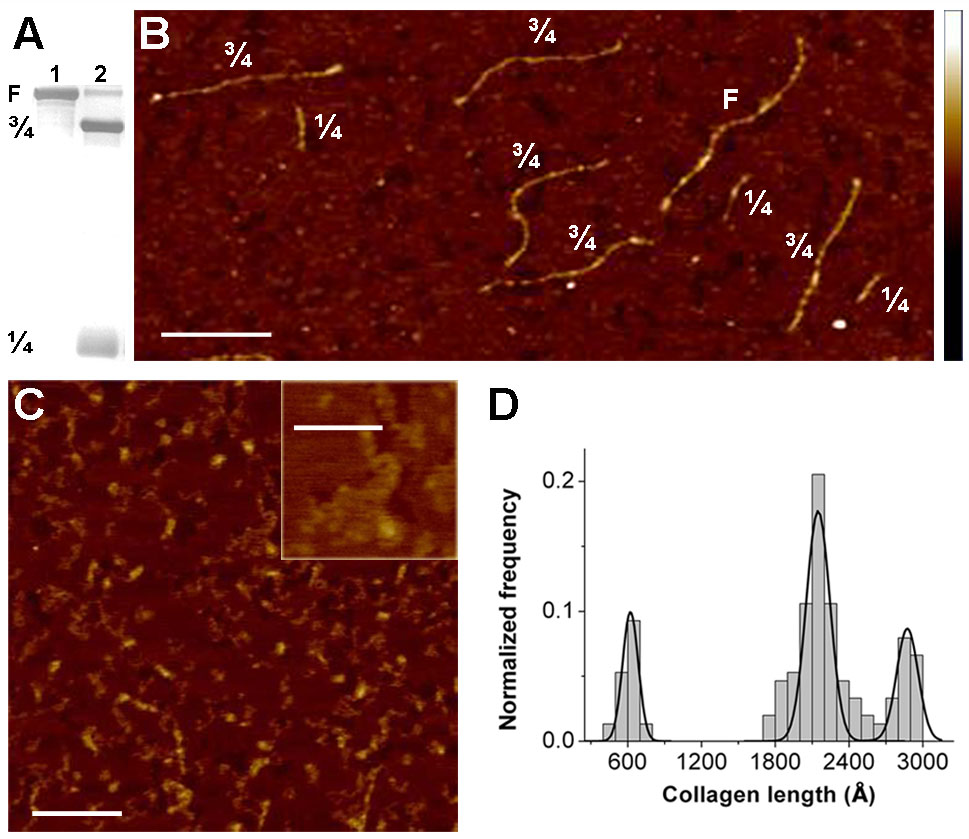


**1000 Å**

**1000 Å**

**500 Å**

Supplement: Figure S3 — Full-length collagen type II cleavage by MMP-8. (A) SDS-PAGE of intact collagen (lane 1, marked F), and MMP-8 produced collagen fragments (lane 2, marked 3/4 and 1/4). Proteolysis of collagen type II by MMP-8 was conducted at 30°C (see supporting Information for collagen thermal stability under these reaction conditions). (B) A mixture of intact and cleaved collagen (1/4 and 3/4 fragments) was adsorbed on mica and scanned by tapping mode. Intact collagen is marked with F and the collagen fragments are marked 1/4 and 3/4. This picture was generated after distinguishing full-length collagen from the 1/4 and 3/4 fragments produced by complete MMP-8 cleavage, in separate AFM experiments. (C) The MMP-8 treated collagen was heat-denatured at 70°C showing complete loss of structural features. Insert - magnification of denatured collagen. Height scale for all images is indicated by the bar to the right, in which the Z-axis ranges from 0 to 20 Å (dark to light). (D) End-to-end collagen length histogram of intact, 3/4 and 1/4 fragments (mean lengths 2820, 2130 and 586 Å, respectively). The values were extracted from the AFM images (n = 151). The Y-axis is the normalized frequency obtained by dividing the counts by the total population. (0.18 MB DOC) [file pone.0011043.s004.doc]

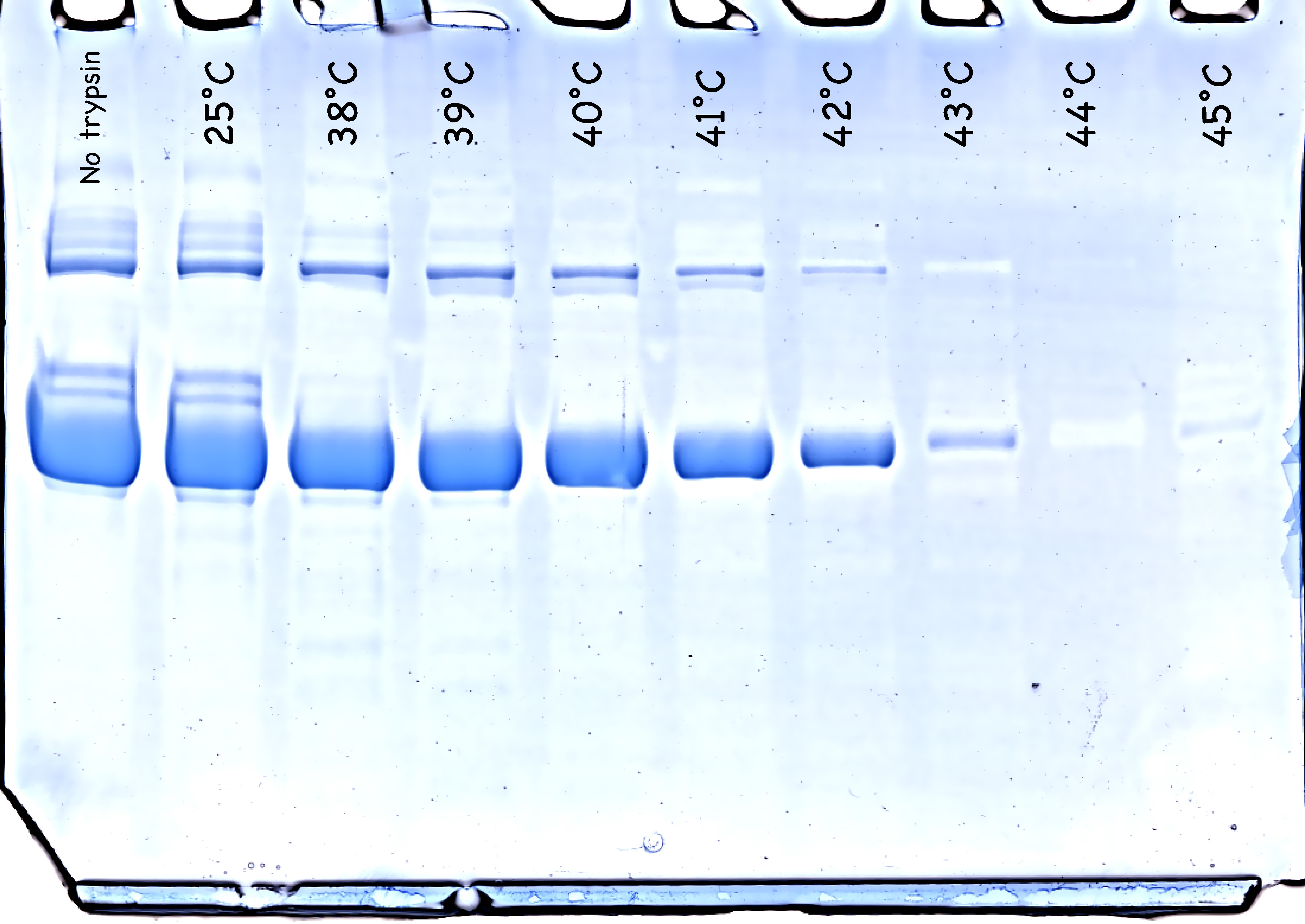

Supplement: Figure S5 — Thermal stability of intact collagen. Since the stability of collagen type II preparations may vary, we include here the collagen thermal stability test assay of the original intact collagen type II preparation used for the AFM experiments. 50 µg of type II collagen (1 mg/ml in digest buffer) incubated at the given temperature for 1 minute, followed by a 4 minute digest with 1 µg of trypsin. The reaction is then stopped with 5 µg of trypsin inhibitor plus 1 µl of 0.5 M EDTA. The digest is mixed with Laemmli buffer and run on a 7.5% gel. This collagen preparation is stable up to 42°C, as indicated by persistence of the bands. (0.86 MB DOC) [file pone.0011043.s006.doc]

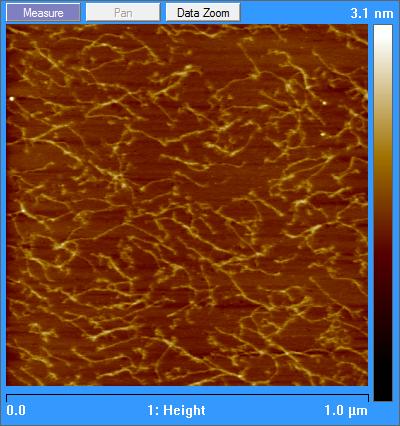


**3000Å**

Supplement: Figure S6 — AFM control image of collagen type II fragment (treated with MMP-8) after 1 hour incubation at 37°C. The collagen fragments retain their intact structure as can be clearly detected by this image. Substantial collagen deformation could be detected only after incubation with MMP-9 as reported in text. (0.06 MB DOC) [file pone.0011043.s007.doc]

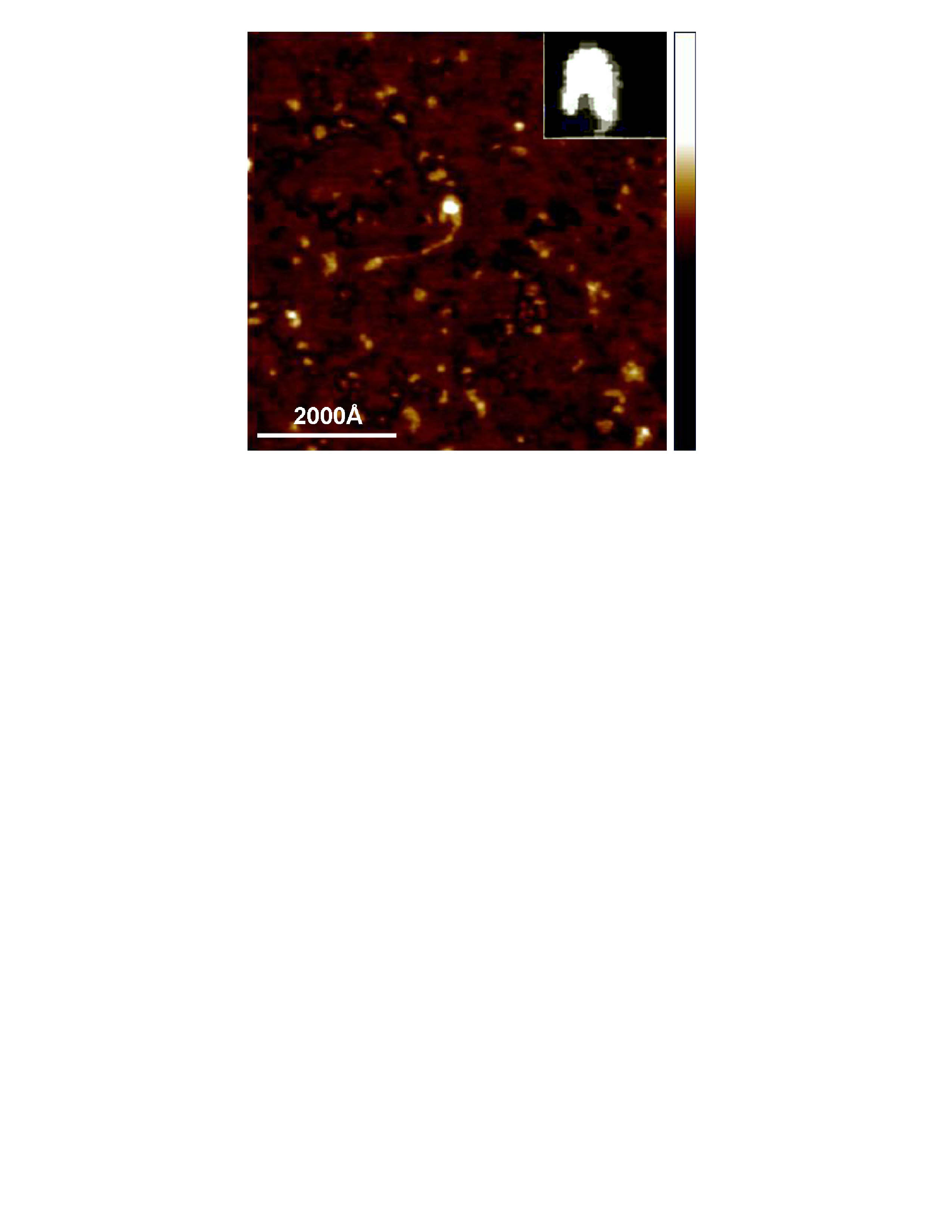

Supplement: Figure S7 — AFM image of 3/4 collagen II fragment treated with MMP-9 for 5 minutes. The height scale is indicated by the bar to the right, in which the Z-axis ranges from 0 to 50 Å (dark to light). Insert - the selected unraveled part of collagen is shown at the right top corner of the image. Magnification 2× of the full presented AFM image. (0.36 MB DOC) [file pone.0011043.s008.doc]

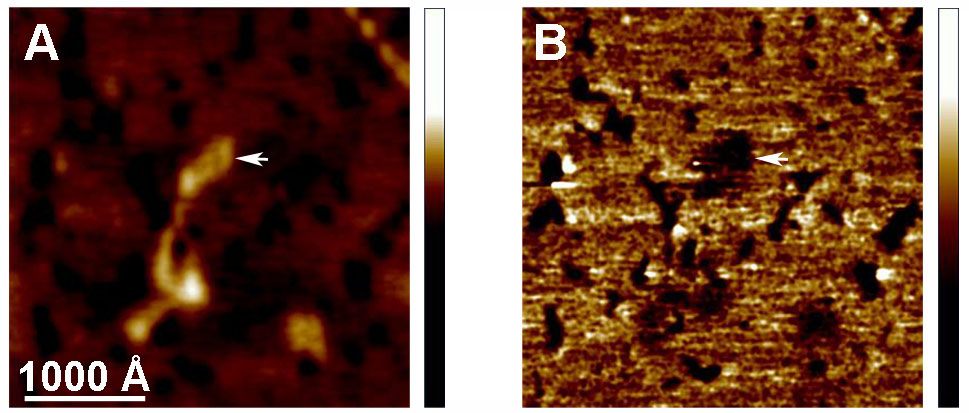

Supplement: Figure S8 — Simultaneously acquired height and phase AFM images of MMP-8-cleaved collagen fragment. (A) Height image of a collagen fragment shows that the upper tail (see arrow) is broadened. The height scale is indicated by the bar to the right, in which the Z-axis ranges from 0 to 3.5 Å (B) The phase image shows that this area appears darker (see arrow). The height scale is indicated by the bar to the right, in which the Z-axis ranges from 0 to 4 degrees. (0.14 MB DOC) [file pone.0011043.s009.doc]

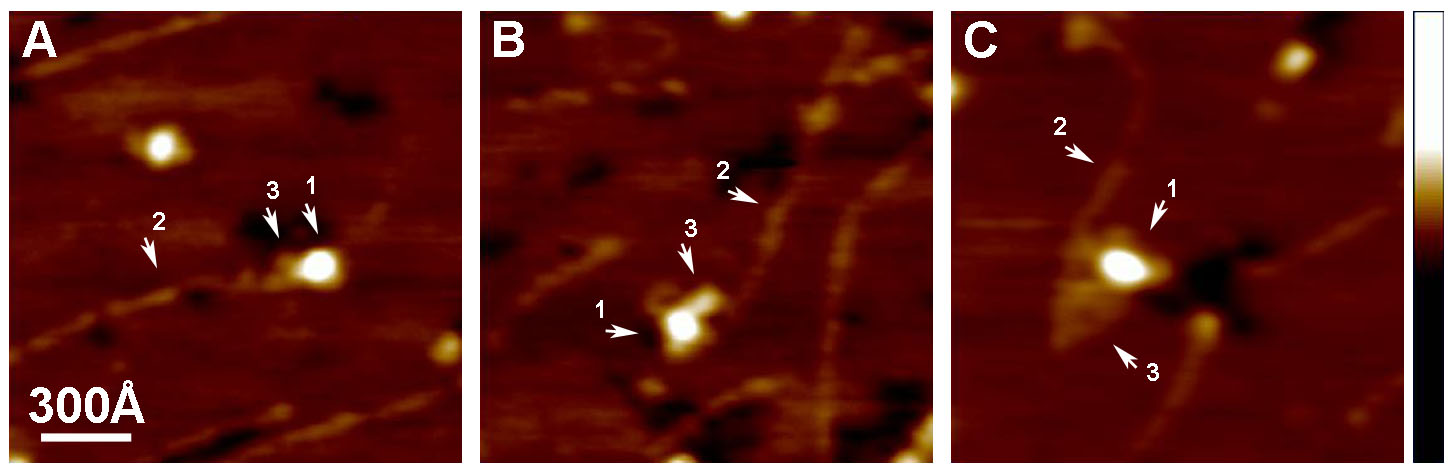

Supplement: Figure S9 — Unwinding of mica-adsorbed collagen II fragments by MMP-9. MMP-8-treated collagen II fragments (arrow 2) were adsorbed directly on mica, supplemented with activated MMP-9 (arrow 1) and inspected for unwinding activity (arrow 3). The different stages of the reaction are shown by representative images: (A) tail binding, (B) tail unwinding (C) gross tail unwinding. The samples were scanned using tapping mode AFM. The ability of MMP-9 to unwind insoluble collagen tails when adsorbed to the mica surface is clearly demonstrated. Height is indicated by the bar to the right, in which the Z-axis ranges from 0 to 40 Å. (0.12 MB DOC) [file pone.0011043.s010.doc]

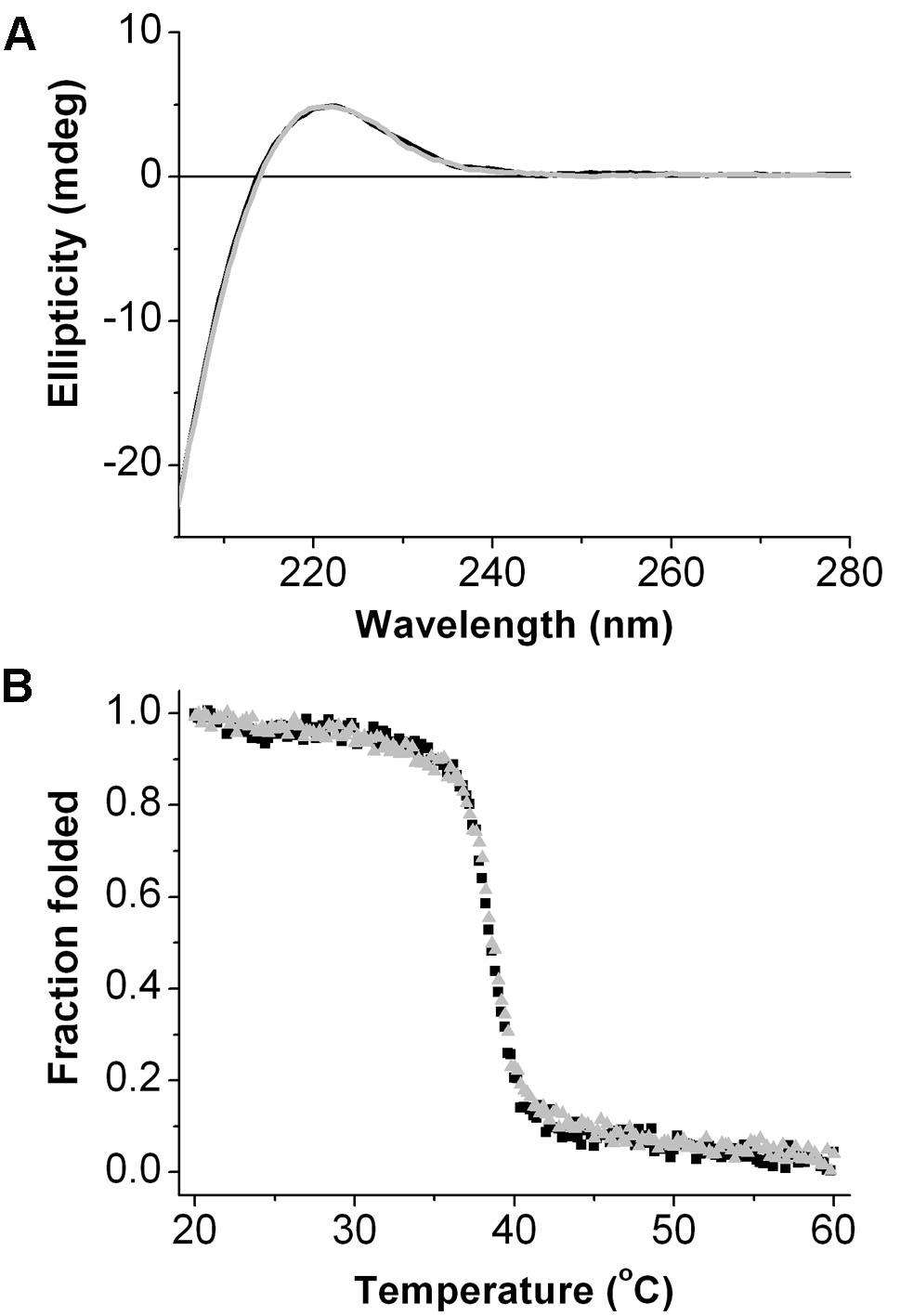

Supplement: Figure S10 — Circular Dichroism spectra and thermal transition curves of MMP-8-cleaved collagen (black), and MMP-8-cleaved collagen supplemented with the MMP-9 inactive mutant MMP-9(E402A) (gray). (A) CD spectra at a collagen concentration of 60 µg/ml shows the unique triple helical signature at 221 nm with no detectable change between the two samples. (B) Thermal transition curves were recorded at 221 nm while the temperature was increased from 20 to 60°C at 0.2°C/min. The differences in melting temperatures, Tm50, are insignificant with 38.5°C between both the MMP-8-cleaved collagen (black), and MMP-8-cleaved collagen supplemented with MMP-9(E402A) (gray). (0.15 MB DOC) [file pone.0011043.s011.doc]

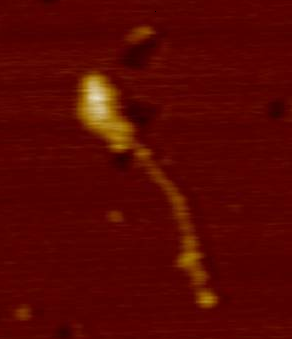

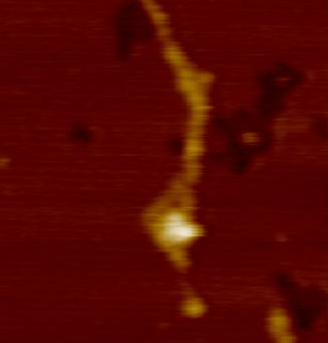

Supplement: Figure S11 — Local denaturating activity of collagen type II fragment by the latent MMP-9(E402A) mutant at 10 minutes incubation time. Right panel shows statistical analysis of the deformed collagen width interfacing with MMP-9 (Black is wild type MMP-9 and gray is MMP-9(E402A) latent mutant), which scales with the total deformed area. Comparison of the mean width with that of the wild type after 10 minutes incubation time reveals that the area resulting from interaction with mutant is approximately 70% that for the wild type. Local collagen deformation by MMP-9(E402A) is significant but less than observed for the wild type enzyme (n = 97 and 23 for the wild type and MMP-9(E402E), respectively). The left panel of the Figure shows images of collagen type II unwound by MMP-9(E402A) (see also Fig. 3E in main text). (0.18 MB DOC) [file pone.0011043.s012.doc]
