# Supplementary material for: Direct Visualization of Protease Action on Collagen Triple Helical Structure
Source: PLoS One. 2010 Jun 16;5(6):e11043. doi: 10.1371/journal.pone.0011043 (PMC2886829; doi:10.1371/journal.pone.0011043)
Supplement: Supplementary file 3 [file pone.0011043.s005.doc]

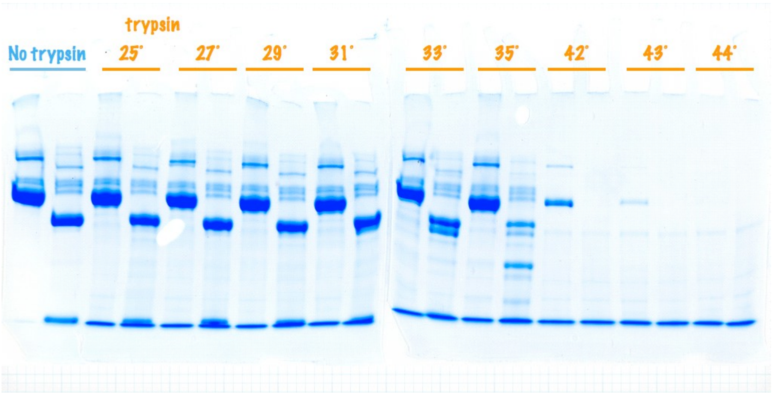
trypsin
No trypsin
25°
27°
29°
31°
33°
35°
42°
43°
44°

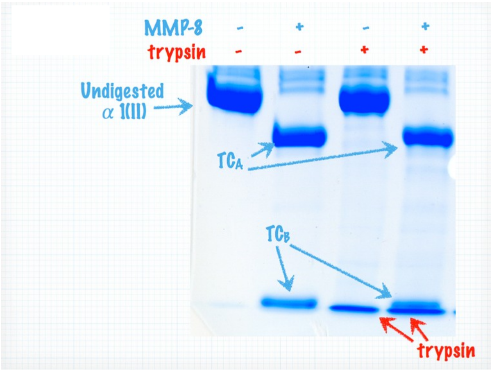
MMP-8 - + - +
trypsin - - + +
Undigested
α 1(II)
TC_A
TC_B
trypsin
